# Lung function trajectories in patients with idiopathic pulmonary fibrosis

**DOI:** 10.1186/s12931-023-02503-5

**Published:** 2023-08-24

**Authors:** Megan L Neely, Anne S Hellkamp, Shaun Bender, Jamie L Todd, Timothy Liesching, Tracy R Luckhardt, Justin M Oldham, Rishi Raj, Eric S White, Scott M Palmer

**Affiliations:** 1 Duke Clinical Research Institute, Durham, NC USA; 2 Duke University Medical Center, Durham, NC USA; 3 Boehringer Ingelheim Pharmaceuticals, Inc, Ridgefield, CT USA; 4 Lahey Hospital & Medical Center, Burlington, MA USA; 5 Department of Medicine, University of Alabama at Birmingham, Birmingham, AL USA; 6 Division of Pulmonary and Critical Care Medicine, University of Michigan, Ann Arbor, MI USA; 7 Stanford University School of Medicine, Stanford, CA USA

**Keywords:** Interstitial lung disease, Lung function testing, Forced vital capacity

## Abstract

**Background:**

Idiopathic pulmonary fibrosis (IPF) is a progressive fibrosing interstitial lung disease characterised by decline in lung function. We evaluated trajectories of forced vital capacity (FVC) and diffusing capacity (DLco) in a cohort of patients with IPF.

**Methods:**

Patients with IPF that was diagnosed or confirmed at the enrolling centre in the previous 6 months were enrolled into the IPF-PRO Registry between June 2014 and October 2018. Patients were followed prospectively, with lung function data collected as part of routine clinical care. Mean trajectories of FVC and DLco % predicted in all patients and in subgroups by characteristics assessed at enrolment were estimated using a joint model that accounted for factors such as disease severity and visit patterns.

**Results:**

Of 1002 patients in the registry, 941 had ≥ 1 FVC and/or DLco measurement after enrolment. The median (Q1, Q3) follow-up period was 35.1 (18.9, 47.2) months. Overall, mean estimated declines in FVC and DLco % predicted were 2.8% and 2.9% per year, respectively. There was no evidence that the mean trajectories of FVC or DLco had a non-linear relationship with time at the population level. Patients who were male, white, had a family history of ILD, were using oxygen, or had prior/current use of antifibrotic therapy at enrolment had greater rates of decline in FVC % predicted. Patients who were male or white had greater rates of decline in DLco % predicted.

**Conclusions:**

Data from the IPF-PRO Registry suggest a constant rate of decline in lung function over a prolonged period, supporting the inexorably progressive nature of IPF. A graphical abstract summarising the data in this manuscript is available at: https://www.usscicomms.com/respiratory/IPF-PRORegistry_LungFunctionTrajectories.

**Trial registration:**

NCT01915511.

**Supplementary Information:**

The online version contains supplementary material available at 10.1186/s12931-023-02503-5.

## Background

Idiopathic pulmonary fibrosis (IPF) is a progressive fibrosing interstitial lung disease (ILD) associated with high mortality [1]. In patients with IPF, decline in lung function reflects disease progression and is predictive of mortality [2–5]. Change in forced vital capacity (FVC) has become established as the primary endpoint in trials of drugs for IPF [6–8]. Observational studies have suggested that most patients with IPF have a near-linear decline in FVC, while some have periods of faster decline, or suffer from acute deteriorations in lung function, termed acute exacerbations [4, 9–12]. Although some studies have suggested that factors such as the extent of fibrosis on high-resolution computed tomography (HRCT) or baseline lung function may impact the rate of decline in FVC or diffusing capacity of the lungs for carbon monoxide (DLco), for an individual patient, the course of IPF remains difficult to predict [13–16].

The Idiopathic Pulmonary Fibrosis Prospective Outcomes (IPF-PRO) Registry (NCT01915511) is an observational US registry of patients with IPF [17]. One of the aims of the registry is to improve understanding of the course of IPF. We used data from this registry to model and evaluate trajectories of FVC and DLco. We also investigated whether patient characteristics at baseline influenced the trajectories of FVC and DLco.

## Methods

### Patients

Patients with IPF that was diagnosed or confirmed at the enrolling centre in the previous 6 months were enrolled into the IPF-PRO Registry at 46 sites between June 2014 and October 2018. At enrolment, retrospective data were taken from patients’ medical records. Patients were then followed prospectively, with lung function data collected as part of routine clinical care. Approximately every 6 months until death, lung transplant, or withdrawal from the registry, lung function measures collected during the prior 6 months were extracted from the patient’s medical record.

### Lung function measurements

Data for this analysis were extracted from the database in September 2021. The analysis cohort included patients who had ≥ 1 FVC or DLco measurement at enrolment or during follow-up. As the data were collected during routine clinical care, the lung function data collected during follow-up varied in the frequency and timing of their collection. The number and timing of FVC and DLco measurements were assessed in descriptive analyses. The baseline measurement was taken between 30 days before and 30 days after enrolment. If a patient had > 1 measurement in that period, the measurement closest to enrolment was used. FVC and DLco % predicted values over 48 months were described in subgroups based on the time from enrolment to a terminal event (no event, ≤ 1 year, > 1 to ≤ 2 years, > 2 to ≤ 3 years, > 3 years) with the trajectories plotted using cubic functions fit through daily means. A terminal event was defined as death, lung transplant, entry into hospice care, or withdrawal from the registry due to worsening of IPF.

### Modelling lung function trajectories

The methodology used in the modelling is described in detail in Additional File 1. The trajectories of FVC and DLco % predicted were modelled using a joint model [18]. This joint model comprised three sub-models, linked by common random effects: (i) a linear mixed-effects model for lung function values, (ii) a frailty model for measurement frequency, and (iii) a proportional hazards model for terminal events. This approach was dictated by two considerations: firstly, that the frequency of lung function testing may be related to patients’ health status (i.e., sicker patients may have pulmonary function tests more frequently) and secondly, that the trajectories of lung function in patients who left the registry due to a terminal event may be different from those who left the registry for other reasons. We used polynomial terms of time to investigate whether the trajectories were linear or non-linear with respect to time and patient-level random intercepts and slopes to examine whether the trajectories were similar among patients.

The following covariates (measured at enrolment) were included in the model: age, sex, race/ethnicity, body mass index (BMI), family history of ILD, diagnostic criteria [19], diagnosis of IPF prior to referral to the enrolling centre, oxygen use with activity and at rest, oxygen use with activity only, prior/current use of antifibrotic therapy (nintedanib or pirfenidone), ever smoked, obstructive sleep apnoea. Missing data for these covariates were imputed using the Full Conditional Specification method. As trajectories of FVC or DLco % predicted may vary among patients with different demographics, disease severity, or medical history, when fitting the models, we looked for interaction between each covariate and time. P-values are presented for all interaction tests and, where the interactions were not statistically significant, for main effects tests.

To illustrate the results, we estimated mean values of FVC and DLco % predicted, for all patients and for the subgroups, at each day of follow-up. We then calculated the differences between the means of the subgroups at baseline (trajectory intercept) and over 1 year (trajectory slope). For covariates that had a significant interaction with time, we present the differences in the trajectory intercept between subgroups and the slopes for each subgroup. For covariates that did not have a significant interaction with time, we present the differences in the intercept between subgroups, along with the slope for the overall cohort. Subgroup trajectories are also shown graphically. To assess whether the trajectories of FVC or DLco % predicted varied among patients with different lung function values at baseline, a further analysis was conducted based on a modified joint model, where post-enrolment lung function tests were used as the outcome and lung function tests at enrolment as one of the predictors.

FVC % predicted values were calculated using equations published by the European Respiratory Society Global Lung Function Initiative [20]. DLco % predicted values were calculated using standard formulas [21, 22] and corrected for haemoglobin. Analyses were conducted using SAS version 9.4 or higher (SAS Institute, Cary, North Carolina, USA).

## Results

### Patients

Of 1002 patients enrolled in the registry, 941 patients had ≥ 1 FVC and/or ≥ 1 DLco measurement at enrolment or during follow-up (940 had ≥ 1 FVC measurement; 901 had ≥ 1 DLco measurement) and comprised the analysis cohort. Their baseline characteristics are shown in Table 1. At baseline, median (Q1, Q3) FVC was 72.9 (62.1, 84.2) % predicted and median (Q1, Q3) DLco was 42.5 (33.0, 51.9) % predicted.

**Table 1:** Baseline characteristics of analysis cohort (n = 941)

Age, years	70 (65, 75)
Male	698 (74)
Body mass index, kg/m^2^	28.9 (26.1, 32.3)
Smoking status	
Former	613 (65)
Never	312 (33)
Current	16 (2)
Race/ethnicity	
White non-Hispanic	787 (92)
Black non-Hispanic	13 (2)
Other non-Hispanic	25 (3)
Hispanic	34 (4)
Diagnostic criteria*	
Definite	613 (65)
Probable	238 (25)
Possible	90 (10)
Diagnosis of IPF prior to referral to enrolling centre	414 (44)
Family history of ILD (in parent, sibling, or grandparent)	175 (20)
Hospitalisation in prior 12 months	257 (28)
Respiratory hospitalisation in prior 12 months	157 (17)
Distance to enrolling centre, miles	33 (14, 93)
Private insurance	568 (60)
FVC % predicted	72.9 (62.1, 84.2)
DLco % predicted	42.5 (33.0, 51.9)
Oxygen use at rest	178 (19)
Oxygen use with activity	315 (33)
Use of antifibrotic therapy	485 (52)
Gastro-oesophageal reflux disease	674 (72)
Coronary artery disease	274 (29)
Obstructive sleep apnoea	262 (28)
Emphysema or chronic bronchitis	144 (15)
Lung or other cancer	67 (7)
Pulmonary hypertension	65 (7)

Data are n (%) or median (Q1, Q3). Not all patients provided data for all variables

*According to 2011 ATS/ERS/JRS/ALAT diagnostic guidelines [19]

### FVC and DLco % predicted over time

The median (Q1, Q3) follow-up period was 35.1 (18.9, 47.2) months. The median (Q1, Q3) times from enrolment to the last FVC and DLco measurements were 21.6 (7.8, 36.3) months and 20.7 (6.3, 35.6) months, respectively. FVC and DLco measurements were reasonably well distributed throughout the follow-up period, although the number of measurements collected per time point decreased over time (Additional Files 2 and 3). The median (Q1, Q3) numbers of FVC and DLco measurements per patient were 4 (2, 8) and 4 (2, 7), respectively. The maximum number of measurements for a single patient was 26 for FVC and 20 for DLco. The median (Q1, Q3) time between measurements was 4.3 (3.0, 6.3) months for FVC and 4.7 (3.2, 6.6) months for DLco. Patients who had shorter times to a terminal event had lower FVC and DLco % predicted values at enrolment (Additional Files 4 and 5).

### Modelling of FVC % predicted over time

**Fig. 1:**
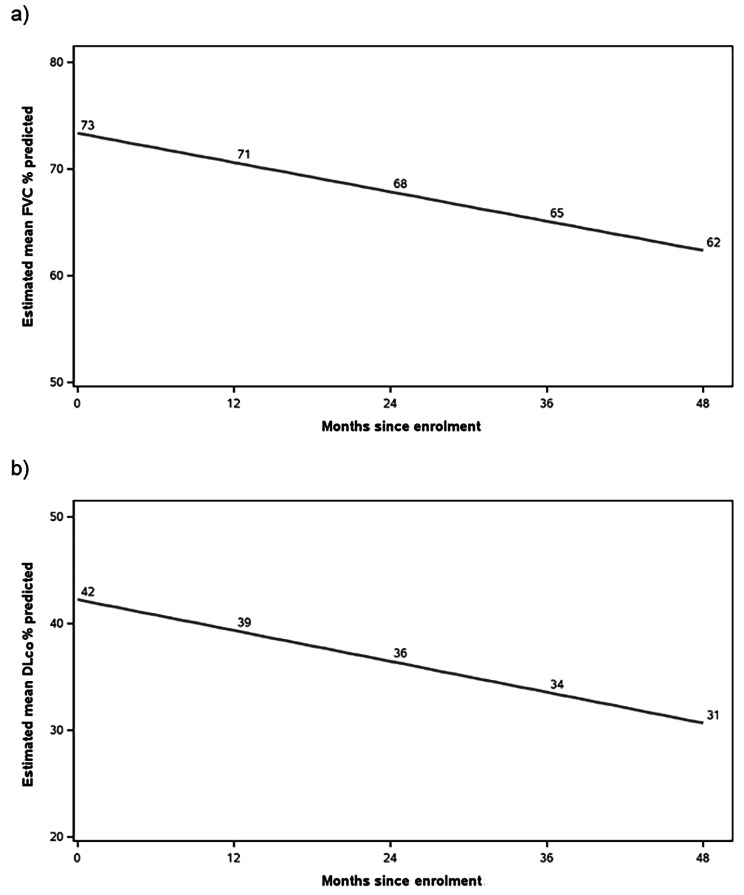
Modelling of (**a**) estimated mean FVC % predicted values over time and (**b**) estimated mean DLco % predicted values over time

The estimated mean decline in FVC was 2.8% predicted per year (Fig. 1a). There was no evidence that the trajectory of FVC had a non-linear relationship with time. Patients with a higher FVC % predicted at baseline had a greater rate of decline in FVC % predicted during follow-up (interaction with time p = 0.0003). Among patients with baseline FVC values of 60%, 70% and 80% predicted, the estimated declines in FVC were 2.5%, 2.7% and 2.9% predicted per year.

**Fig. 2:**
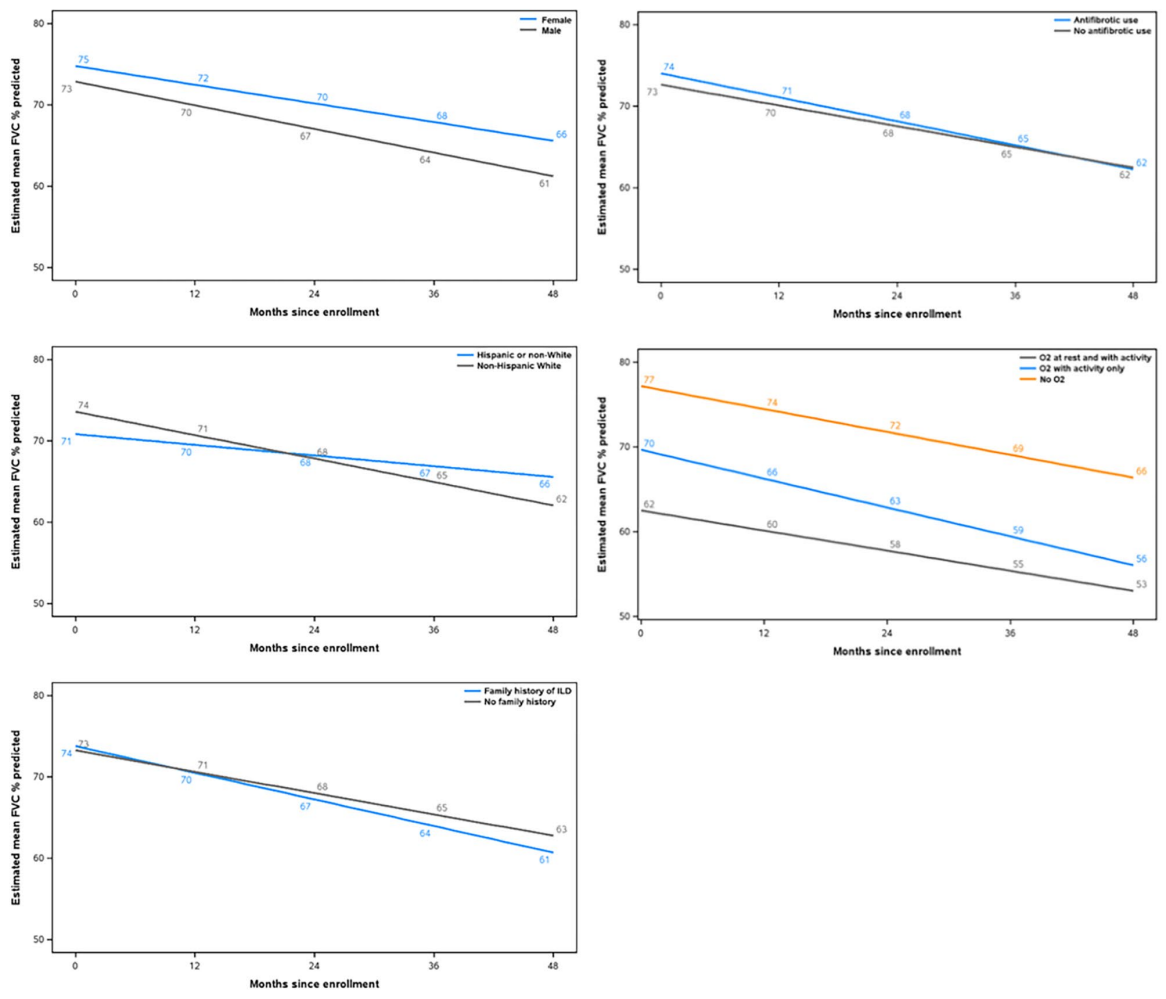
Modelling of estimated mean FVC % predicted values in subgroups with significant interactions with time

Oxygen use, sex, race/ethnicity, family history of ILD, and use of antifibrotic therapy showed significant interactions with time. Specifically, patients who were male, white, had a family history of ILD, were using oxygen (with activity and at rest or with activity alone) or had prior/current use of antifibrotic therapy had greater rates of decline in FVC % predicted (Additional File 6; Fig. 2).

**Fig. 3:**
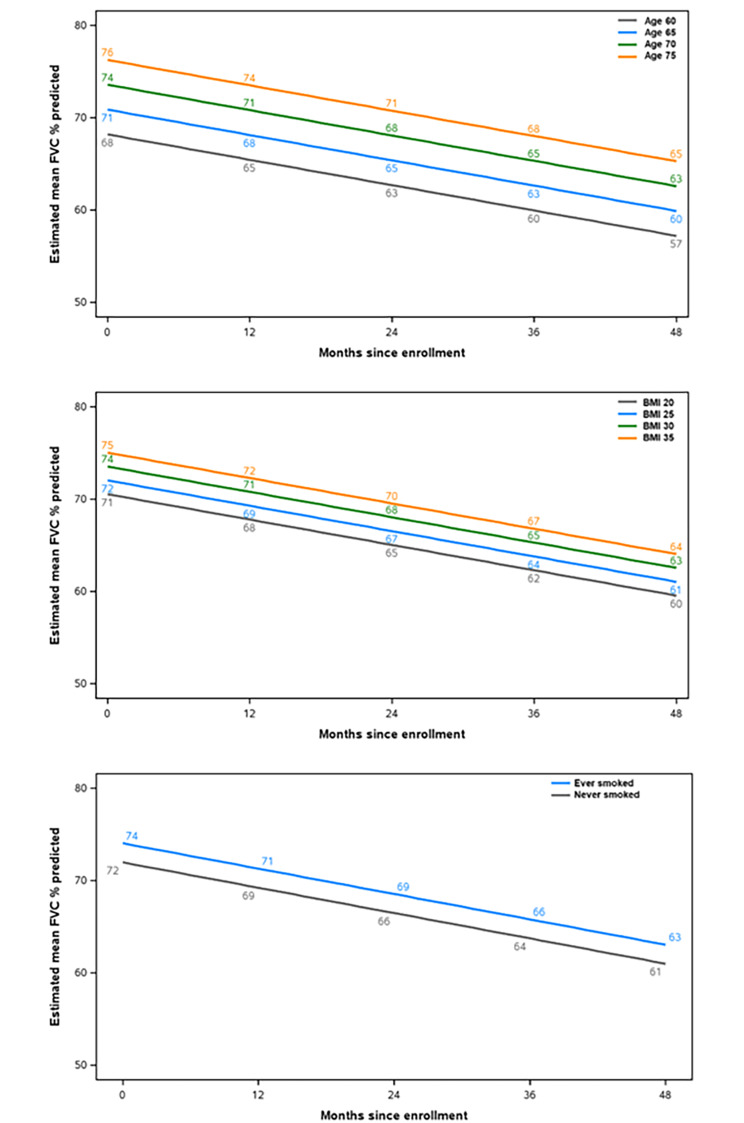
Modelling of estimated mean FVC % predicted values in subgroups with significant associations with FVC % predicted at baseline but without significant interactions with time

Age, BMI, and smoking status were significantly associated with FVC % predicted at baseline but did not have significant interactions with time. Specifically, older patients, those with a higher BMI and current or former smokers had higher baseline values of FVC % predicted, but similar rates of decline (Additional File 6; Fig. 3).

### Modelling of DLco % predicted over time

The estimated mean decline in DLco was 2.9% predicted per year (Fig. 1b). There was no evidence that the trajectory of DLco had a non-linear relationship with time. The rate of decline in DLco % predicted was similar irrespective of the baseline value (interaction with time p = 0.60).

**Fig. 4:**
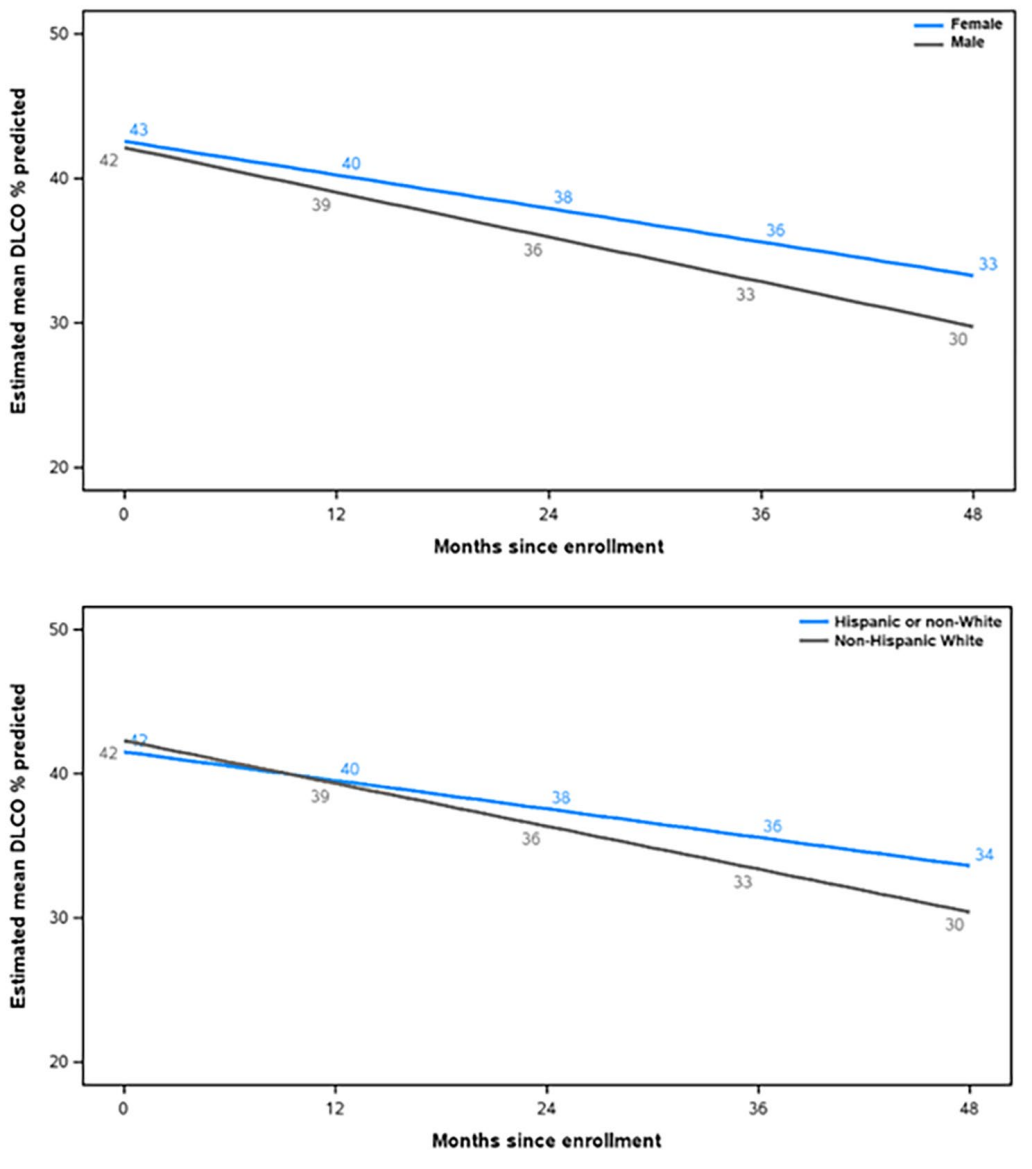
Modelling of estimated mean DLco % predicted values in subgroups with significant interactions with time

Sex and race/ethnicity showed significant interactions with time. Patients who were male or white had a greater rate of decline in DLco % predicted (Additional File 7; Fig. 4).

**Fig. 5:**
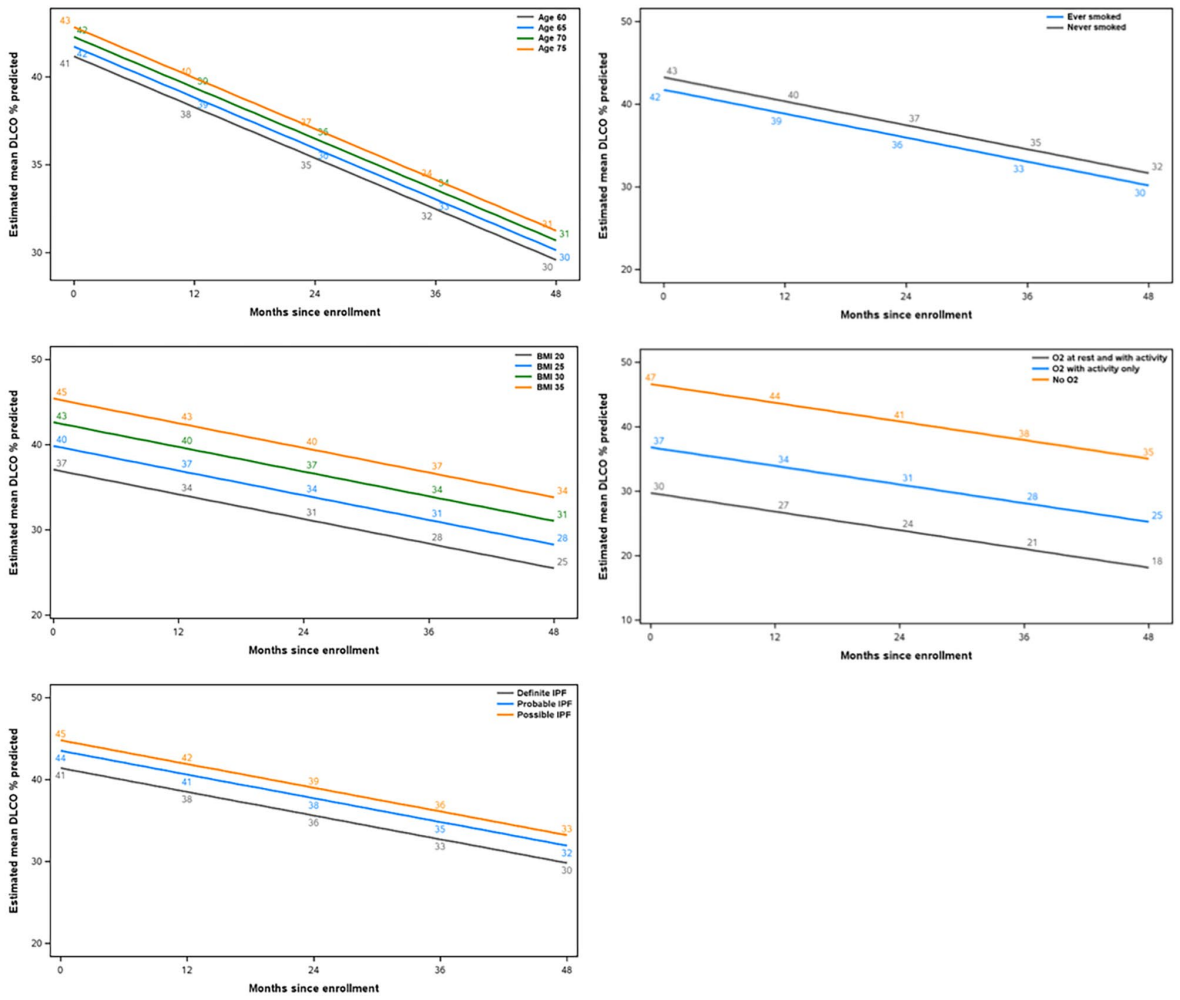
Modelling of estimated mean DLco % predicted values in subgroups with significant associations with DLco % predicted at baseline but without significant interactions with time. Definite, probable and possible IPF according to 2011 ATS/ERS/JRS/ALAT diagnostic guidelines [19]

Age, BMI, oxygen use, smoking status and diagnostic criteria were significantly associated with DLco % predicted at baseline, but did not show significant interactions with time. Specifically, older patients, those with a higher BMI, never smokers, those not using oxygen, and those with possible IPF had higher baseline values of DLco % predicted, but similar rates of decline (Additional File 7; Fig. 5).

### Shared random effects and associations between sub-models in the joint model

The random effects shared among the models for both lung function measures indicated that there was significant heterogeneity in the number of lung function tests per patient (FVC: σ^2^_u_ = 0.53; DLco: σ^2^_u_ = 0.45; both p < 0.0001) and in the values at each assessment (FVC: σ^2^_v_ = 163.88; DLco: σ^2^_v_ = 88.19; both p < 0.0001). The associations between the sub-models of each joint model were significant. The association between the number of lung function tests and their values was negative, suggesting that as lung function declined, the frequency of testing increased (FVC: γ_1_ = − 14.05; DLco: γ_1_ = − 10.44; both p < 0.0001). The association between the number of lung function tests and a terminal event was positive, suggesting that as the frequency of testing increased, so did the risk of a terminal event (FVC: γ_2_ = 5.73; DLco: γ_2_ = 5.78; both p < 0.0001). The association between the values of lung function tests and a terminal event was negative, suggesting that as lung function declined, the risk of a terminal event increased (FVC: γ_3_ = − 0.033, p = 0.04; DLco: γ_3_ = − 0.050, p = 0.02).

## Discussion

We used data from the IPF-PRO Registry to investigate the trajectories of lung function decline, and potential predictors of faster decline, in a cohort of patients with IPF. Based on a joint model that adjusted for factors such as demographics, disease severity, and visit patterns, the trajectories of FVC and DLco % predicted appeared to indicate constant rates of decline over time. The significant shared random effects across the models for both FVC and DLco demonstrated that there were relationships among the frequency of measurements, the values, and the occurrence of terminal events indicating disease progression. Specifically, the relationships suggest that a decline in lung function is associated with an increased frequency of testing and an increased risk of a terminal event. Given this, the use of standard repeated measures models, such as linear mixed modelling, would not have been appropriate for estimating the trajectories (i.e., the assumptions underlying these models would not have been fulfilled); a joint modelling approach was required.

Caution should be employed in comparing the findings of studies that used different methodologies, but the estimated annual decline in FVC % predicted in our analyses (2.8%) was similar to that reported in other observational studies in patients with IPF (3.5% and 4.4%) [9, 23]. In the placebo groups of clinical trials in patients with IPF, mean declines in FVC % predicted of 6.1–7.2% have been reported over 48 to 52 weeks [7, 24–26]. It should be noted that the joint model used in our analysis estimated the mean trajectory within the population, and that there is heterogeneity in patient-specific trajectories. While we found no evidence of a non-linear relationship with time at the population level, the observed trajectories for some patients had a non-linear trend over time. In an analysis of data from the PROFILE study conducted in 451 patients with IPF, machine learning methods identified four FVC trajectories: near-linear decline over 3 years (34% of patients), initial decline and then stabilisation (27% of patients), initial improvement and then decline (24% of patients), and stability (15% of patients) [27].

Patients with shorter times to a terminal event had lower FVC % predicted and DLco % predicted values at enrolment. This was unsurprising given the known association between worse lung function and the risk of hospitalisation or death in patients with IPF [4, 28–32]. In prior analyses of data from the IPF-PRO Registry, lower FVC % predicted at enrolment was associated with an increased risk of death or lung transplant during 30 months of follow-up with a hazard ratio of 1.28 (95% CI: 1.10, 1.49) per 10% lower FVC % predicted [29]. Interestingly, at baseline, FVC % predicted was lower in younger patients. While this may seem counterintuitive, our previous analysis showed that the youngest patients had the highest risk of death or lung transplant during follow-up [29]. This may reflect familial disease, as a greater proportion of the younger participants had a family history of ILD (27% of those aged < 65 years versus 12% of those aged ≥ 75 years) and familial pulmonary fibrosis is known to be associated with high mortality [33]. Patients with a history of smoking also had a higher FVC % predicted at baseline, likely reflecting the higher prevalence of emphysema in these patients [34].

The course of FVC decline in patients with IPF is challenging to predict and regular measurement of lung function is an important element of management. The latest ATS/ERS/JRS/ALAT clinical practice guideline recommends pulmonary function testing approximately every 4 to 6 months, or sooner if clinically indicated [1]. Our findings suggest that patients with a higher FVC at baseline had a greater rate of decline in FVC % predicted over the follow-up period. Previous studies have provided conflicting results: some have reported differing rates of FVC decline among patients with different baseline values [16, 32], while others have reported no association [23, 35–38]. In our analyses, patients who were male had greater rates of decline in FVC and DLco % predicted during follow-up. Previous studies have also found that male patients with IPF have poorer outcomes than female patients, but the reason for this is unclear [23, 39–42]. Our observation that white non-Hispanic patients had greater rates of lung function decline should be interpreted with caution given that only a small number of patients in the cohort were Hispanic or non-white (n = 154). Patients who were using supplementary oxygen at baseline had a greater rate of decline in FVC % predicted than those who were not using oxygen. Oxygen use has been associated with FVC decline and mortality in several studies in patients with IPF [16, 43, 44] and may be considered a marker of severe disease and a predictor of poor outcome.

Strengths of our analyses include the large cohort of patients with IPF and the use of joint model that accounted for the irregular frequency of measurements and for potential differences in trajectories of lung function between patients who did and did not have terminal events. However, it should be noted that patients in the IPF-PRO Registry may not be representative of the general population of patients with IPF given that most received their care at specialised centres. Although statistically significant differences in the trajectories of FVC and DLco were observed across several subgroups, in some cases, the actual differences were small. We acknowledge that patients who had more rapidly declining lung function prior to enrolment may have been more likely to have prior/current exposure to antifibrotic therapy at enrolment. As the three-part joint model we used did not allow for the inclusion of time-dependent covariates, the model did not adjust for differences post-enrolment, including starting or stopping antifibrotic therapy, which may have influenced lung function decline.

## Conclusions

These analyses of data from the IPF-PRO Registry suggest that the rate of decline in lung function in the overall population was constant over a prolonged period. Lung function at enrolment and/or the rate of decline in lung function during follow-up differed across subgroups based on clinical factors or demographics. These data support the inexorably progressive nature of IPF.

### Electronic supplementary material

Below is the link to the electronic supplementary material.

Supplementary Material 1

## Data Availability

The datasets analysed during the current study are not publicly available, but are available from the corresponding author on reasonable request.

## List of abbreviations

BMI: body mass index
DLco: diffusing capacity of the lungs for carbon monoxide
FVC: forced vital capacity
HRCT: high-resolution computed tomography
IPF: idiopathic pulmonary fibrosis
ILD: interstitial lung disease
