# Supplementary material for: Lung function trajectories in patients with idiopathic pulmonary fibrosis
Source: Respir Res. 2023 Aug 24;24:209. doi: 10.1186/s12931-023-02503-5 (PMC10463468; doi:10.1186/s12931-023-02503-5)
Supplement: Supplementary file 1 — Supplementary Material 1 [file 12931_2023_2503_MOESM1_ESM.docx]

**Additional files**

**Additional File 1: Statistical Methods**

**Modelling lung function trajectories**

*Approach*

The analysis required consideration of several issues:

1. Lung function data were irregularly collected and the frequency of collection may have been related to patients’ health status. Thus, repeated measures analysis and use of time-dependent covariates could not be used. Repeated measures assumes regular collection points that are independent of patient status, while time-dependent covariates are appropriate only when the values of the variable are known at all times and are measured without error.
2. The lung function trajectories of patients who left the registry due to a terminal event may have been different from those of patients who left the registry for other reasons.
3. While lung function trajectories may share some similarities in shape (slope and intercept) among patients, there may be considerable variation, and the shape of lung function trajectories may vary across subgroups defined by demographics, disease severity, or medical history.
4. The trajectory of lung function may be non-linear over time.

Thus, our analysis approach needed to:

1. Simultaneously consider visit patterns, lung function measurements, and terminal events.
2. Allow the use of random effects for the intercept and slope so that the shape of each patient’s trajectory could be flexible with respect to time.
3. Allow for a non-linear relationship between time and lung function values.
4. Adjust for patient types and examine interactions between patient types and trajectory shapes.

We used the joint modelling approach described by Liu et al [Biometrics 2008;64:950–58]. This joint model included three sub-models (one for measurement frequency, one for lung function values, one for terminal events) linked by common random effects.

*Analyses*

An examination of the relationship between time and FVC % predicted and DLco % predicted showed that a linear fit would adequately describe the relationship for both lung function tests. The lung function models included all specified covariates. The measurement frequency and terminal event models included covariates that were identified as important for each endpoint using a simplified (fixed effects) modelling approach. Age and body mass index were evaluated for the linearity of their relationship with lung function. For both variables and both lung function tests, a linear fit was adequate. Two terms, here referred to as *u* and *v*, were included to capture the random effects shared among the three sub-models within each joint model. The two terms along with their coefficients (γ_1_, γ_2_, γ_3_) allowed estimation of the three possible sharing patterns, *i.e*., lung function–measurement frequency, lung function–terminal event, and measurement frequency–terminal event.

In addition, patient-level random effects for both the intercept and slope of time were assessed. The random effect for intercept was not significant when included in the FVC% predicted model with all covariates (p=0.99). This indicated that the covariates included in the model were adequate to explain patient-to-patient variation in intercept. The random effect for slope was significant when included in the FVC % predicted model with all covariates (p<0.001). However, several interactions between time and covariates were significant in a model that did not include this random effect and no longer significant in a model that did include it. This indicated that these interactions were explaining much of the same patient-to-patient variation in slope as the random effect. As the goal of this analysis was an explanatory model, we opted to retain the interaction terms in the model. A similar pattern was observed with DLco % predicted. Therefore, the patient-level random effects for intercept and slope of the time terms were dropped from further consideration.

To assess the importance and nature of the interactions in the lung function models, we used backward selection. For each lung function parameter, we began with a model that included all possible interactions between time and covariates, and eliminated the interactions, one at a time, from highest to lowest p-value. The process was stopped when all remaining interactions had p<0.05. P-values reported for interactions reflect this process, *i.e.,* for non-significant interactions the p-value was taken from the step at which the decision was made to omit the term; for significant interactions, the p-value was taken from the final model. Parameter estimates and significance tests for covariates that did not have interactions were generated from the full model. The full model was used to generate estimated values for plotting all covariates, as well as estimates and significance tests for the shared random effects. Where a covariate had a significant interaction with time, its estimates were shown at different time points. Where a covariate did not have a significant interaction with time, a single estimate was shown. The overall estimate for time was generated from a model that did not include interactions and can be interpreted as the mean effect across all levels of other covariates. The reported time p-value was from the full model including all time interactions. The overall estimate for time was plotted with measured values for each lung function parameter. Estimated lung function values were calculated for each day overall and within subgroups. We then calculated the difference in the means of the subgroups at baseline (trajectory intercept) and over 1 year (trajectory slope).

*Examination of patterns of trajectories in subgroups defined by patient characteristics*

Covariates that had different intercepts or slopes were further explored. Estimated lung function values were summarised. For each patient, at each time point, an estimated value was generated as if the patient had all their own covariate values except the covariate being summarised; estimations were generated for each value of that covariate. These values were then averaged for each covariate value at each time point. For example, for sex, an estimated value at baseline was generated for each patient in the cohort using all of their covariates except sex and generating the estimated values as if all patients were female. Values were then generated again as if all patients were male. Each of these sets of estimated values were then averaged. All estimated values were generated from the model that included all interactions with time.

*Relationship between baseline lung function and trajectory*

One question of interest was whether trajectories differed depending on lung function at the time of entry into the registry. This required a separate analysis, as the predictor of interest (baseline lung function) was one of the outcome values in the joint model. For each lung function parameter, a modified joint model was used, with the same form as those mentioned above, except that only post-enrolment lung function values were used as the outcome, and enrolment lung function values were used as one of the predictors. This meant that only a subset of patients could be used, as patients who did not have both an enrolment value and at least one follow-up value were omitted. For FVC % predicted, data from 795 of 940 patients were used and for DLco % predicted, data from 717 of 901 patients were used.

We were interested in the interaction between baseline lung function and time, that is, whether the slope differed depending on the initial value. We were not interested in a main effect, *i.e.,* a difference in intercepts, as we would expect the baseline value to be very close to the estimated intercept. Joint models included the lung function-x-time interactions but no other time interactions.

Additional File 2: Figure S1. Density of FVC measurements over follow-up.


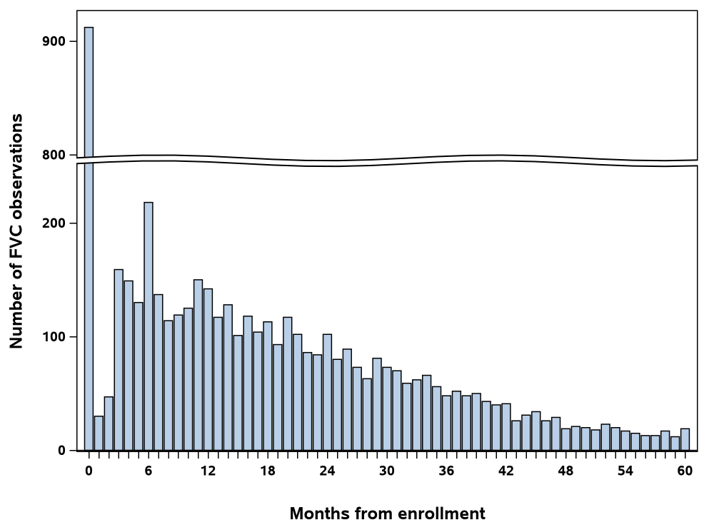


Additional File 3: Figure S2. Density of DLco measurements over follow-up.


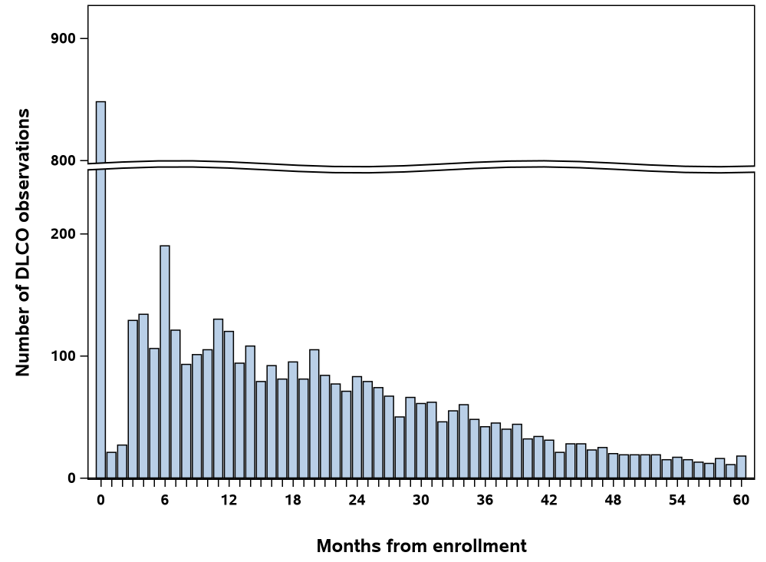


Additional File 4: Figure S3. FVC % predicted values over time.


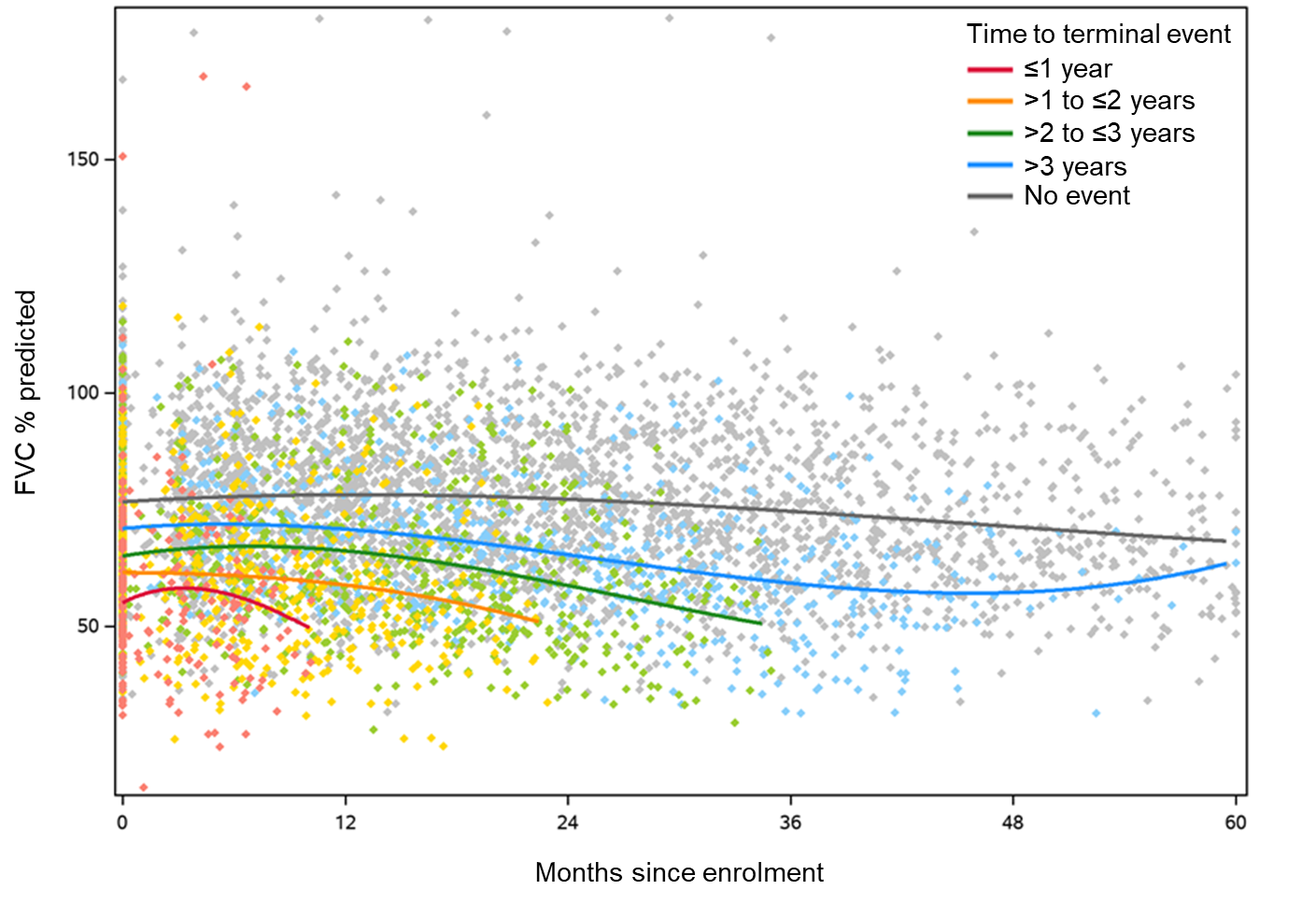


Additional File 5: Figure S4. DLco % predicted values over time.


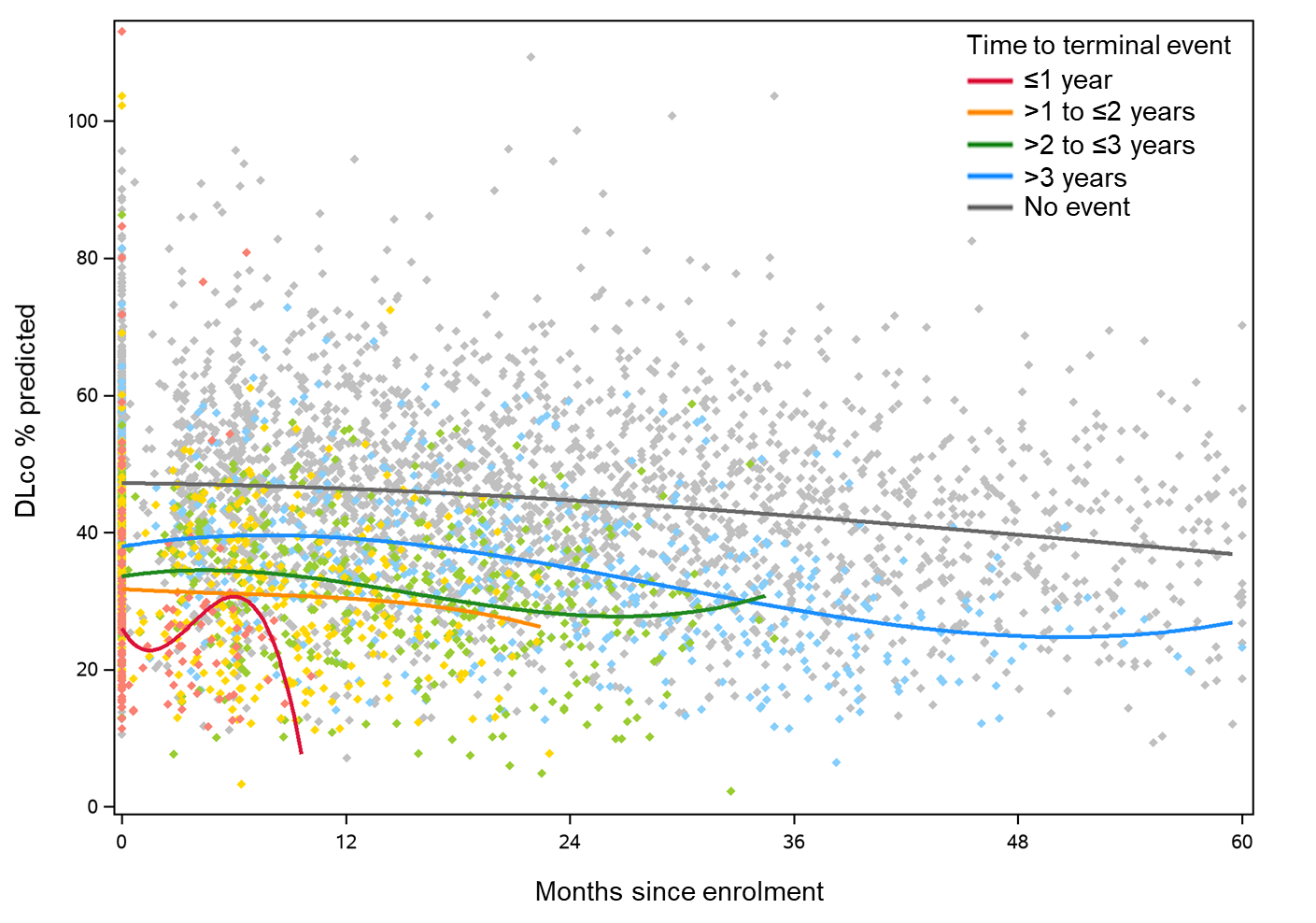


Additional File 6: Table S1. Joint model results for FVC % predicted

| **Covariate** | **P-value for**  **interaction with time (different slopes)** | **P-value for**  **main effect (different intercepts)** | **Difference in estimated mean FVC % predicted at baseline (trajectory intercept)** | **Mean estimated annual rate of change in FVC % predicted (trajectory slope)** |
| --- | --- | --- | --- | --- |
| **With time interaction** |  |  |  |  |
| Sex | 0.0056 | — |  |  |
| Female |  |  | 1.92 | –2.30 |
| Male (reference) |  |  | — | –2.90 |
| Race | 0.0001 | — |  |  |
| Hispanic or non-white race |  |  | –2.75 | –1.31 |
| White race (reference) |  |  | — | –2.88 |
| Family history of ILD | 0.0068 | — |  |  |
| Family history |  |  | 0.52 | –3.27 |
| No family history (reference) |  |  | — | –2.62 |
| Oxygen use | 0.020 | — |  |  |
| Oxygen use with activity and at rest |  |  | –14.66 | –2.37 |
| Oxygen use with activity only |  |  | –7.50 | –3.40 |
| No use (reference) |  |  | — | –2.70 |
| Prior/current use of antifibrotic therapy | 0.039 | — |  |  |
| Use |  |  | 1.40 | –2.94 |
| No use (reference) |  |  | — | –2.54 |
| **Without time interaction** |  |  |  |  |
| Age (per 5 years) | 0.87 | <0.0001 | 2.69 |  |
| Body mass index (per 5 points) | 0.25 | 0.014 | 1.50 | –2.75 |
| Ever smoked (vs never smoked) | 0.56 | 0.031 | 2.06 | All subgroups in this section had the same rate of change, which is the estimated rate in the overall cohort. The difference between subgroups remained constant over time. |
| Diagnostic criteria* | 0.056 | 0.35 |  |  |
| Definite IPF |  |  | 1.69 |  |
| Probable IPF |  |  | 2.41 |  |
| Possible (reference) |  |  | — |  |
| Obstructive sleep apnoea | 0.62 | 0.75 | –0.32 |  |
| Diagnosis of IPF prior to referral to enrolling centre | 0.95 | 0.79 | 0.24 |  |

*According to 2011 ATS/ERS/JRS/ALAT diagnostic guidelines [Am J Respir Crit Care Med 2011;183:788–824].

Additional File 7: Table S2. Joint model results for DLco % predicted.

| **Covariate** | **P-value for**  **interaction with time (different slopes)** | **P-value for**  **main effect (different intercepts)** | **Difference in mean estimated DLco % predicted at baseline (trajectory intercept)** | **Mean estimated annual rate of change in DLco % predicted (trajectory slope)** |
| --- | --- | --- | --- | --- |
| **With time interaction** |  |  |  |  |
| Sex | 0.0011 | — |  |  |
| Female |  |  | 0.44 | –2.32 |
| Male (reference) |  |  | — | –3.09 |
| Race | 0.0301 | — |  |  |
| Hispanic or non-white race |  |  | –0.78 | –1.98 |
| White race (reference) |  |  | — | –2.97 |
| **Without time interaction** |  |  |  |  |
| Oxygen use | 0.2240 | <0.0001 |  |  |
| Oxygen use with activity and at rest |  |  | –16.89 | –2.89 |
| Oxygen use with activity only |  |  | –9.80 | All subgroups in this section had the same rate of change, which is the estimated rate in the overall cohort. The difference between subgroups remained constant over time. |
| No use (reference) |  |  | — |  |
| Body mass index (per 5 points) | 0.6651 | <0.0001 | 2.76 |  |
| Diagnostic criteria* | 0.3058 | 0.0022 |  |  |
| Definite IPF |  |  | –3.39 |  |
| Probable IPF |  |  | –1.28 |  |
| Possible (reference) |  |  | — |  |
| Ever smoked (vs never smoked) | 0.6617 | 0.0429 | –1.50 |  |
| Age (per 5 years) | 0.8777 | 0.0480 | 0.55 |  |
| Obstructive sleep apnoea | 0.7848 | 0.5136 | –0.52 |  |
| Diagnosis of IPF prior to referral to enrolling centre | 0.2826 | 0.5234 | –0.44 |  |
| Family history of ILD (vs no family history) | 0.1281 | 0.8056 | 0.21 |  |
| Prior/current use of antifibrotic therapy (vs no use) | 0.5402 | 0.8263 | 0.15 |  |

*According to 2011 ATS/ERS/JRS/ALAT diagnostic guidelines [Am J Respir Crit Care Med 2011;183:788–824].
